# Ateroprotective effects of *Plinia cauliflora* in. New Zealand rabbits: beyond the lipid-lowering effect

**DOI:** 10.3389/fphar.2024.1244632

**Published:** 2024-01-12

**Authors:** Ana Paula Cestari, Francielly Mourão Gasparotto, Cândida Aparecida Leite Kassuya, Tauany Milan Ribeiro Lacerda, Guilherme Donadel, Catia Sari Moura, Daniela Boleta Ceranto, Ezilda Jacomassi, Odair Alberton, Salviano Belletini Tramontini, Luana Ale Bertoncello, Arquimedes Gasparotto Junior, Emerson Luiz Botelho Lourenço

**Affiliations:** ^1^ Master’s Degree in Medicinal Plants and Herbal Medicines in Basic Healthcare, Paranaense University, Umuarama, Brazil; ^2^ Laboratory of Cardiovascular Pharmacology (LaFaC), Faculty of Health Sciences, Federal University of Grande Dourados, Dourados, Brazil

**Keywords:** anti-inflammatory, antioxidant, atherosclerosis, cardioprotective, jabuticaba

## Abstract

**Introduction:** *Plinia cauliflora* [Mart.] Kausel (Myrtaceae), popularly known as “jabuticaba,” is a fruit species native to Brazil. Despite extensive widespread usage, its antiatherosclerotic properties' impact remains unknown. Thus, the present study aimed to investigate the cardioprotective effects of a preparation obtained from the fruit peels of *P. cauliflora* (EEPC).

**Methods:** Male New Zealand rabbits received a 1% cholesterol-supplemented diet for 60 days. On the thirtieth day, the animals were divided into five experimental groups and received, once a day, by the oral route, the EEPC (10, 30, and 100 mg/kg), simvastatin (2.5 mg/kg), or vehicle for 30 days. At the end of the experimental period, peripheral blood and arterial branch samples were collected. The levels of total cholesterol (TC), low-density lipoprotein cholesterol (LDL-C), high-density lipoprotein cholesterol (HDL-C), triglyceride (TG), malondialdehyde (MDA), nitrotyrosine (NT), nitrite, interleukin 1 beta (IL-1b), interleukin 6 (IL-6), soluble inter-cellular adhesion molecule-1 (sICAM-1), and soluble vascular cell adhesion molecule-1 (sVCAM-1) levels were measured. Moreover, the catalase and superoxide dismutase levels were measured on the arterial samples. Histopathological analysis and arterial morphometry were also performed.

**Results and discussion:** The oral administration of ESEG significantly lowered the levels of lipids in rabbits that were fed a CRD diet. This treatment also adjusted the protective system against oxidation in the arteries by decreasing the oxidation of lipids and proteins. Additionally, the levels of IL-1b, IL-6, sICAM-1, and sVCAM-1 in the bloodstream decreased significantly, and this was accompanied by a reduction of atherosclerotic lesions in all branches of the arteries. The findings suggest that EEPC may be a possible option for additional management of atherosclerosis.

## 1 Introduction

Atherosclerosis has a significant impact on patients’ quality of life. It is characterized by a gradual and silent beginning, which leads to damage to the arterial surface. The initial event of atherogenesis occurs due to a disorder in the function of blood vessel cells, with high levels of cholesterol being the main contributing factor. The presence of the atherosclerotic plaque can cause damage in different parts of the artery and is responsible for complications such as heart attacks, intermittent limb claudication, and strokes (Fan and Watanabe, 2022). Currently, there are many different treatments available for dyslipidemias (abnormal levels of lipids in the blood) and for the prevention of atherosclerosis. Despite their effectiveness, these treatments can have adverse effects on liver function, cause muscle pain, and in rare cases, lead to muscle breakdown. This has encouraged research into alternative treatments, including the use of herbal medicines (Singh, 2019).

*Plinia cauliflora* [Mart.] Kausel (Myrtaceae), popularly known as “jabuticaba,” is a fruit species native to Brazil. Its fruits are traditionally widely used to prepare drinks and jellies (Inada et al., 2015). In traditional medicine, the infusion of leaves and fruit peels has been used to treat asthma, sore throat, and disorders of the gastrointestinal and cardiovascular systems (Giraldi and Hanazaki, 2010).

*Plinia cauliflora* fruit peels present intense violet color from accumulating different polyphenolic pigments, including anthocyanins and ellagic acid derivatives (Neves et al., 2018). A study by Romão et al. (2019) identified 37 compounds in a preparation obtained from *P. cauliflora* fruit peels, including organic acids, phenolic acid derivatives, flavonoids, anthocyanins, and hydrolysable tannins (gallotannins and ellagitannins).

Several preclinical pharmacological studies have suggested *P. cauliflora* as a promising species for treating cardiovascular diseases. Previous data have reported antioxidant (Leite et al., 2011; Hsu et al., 2016), hypotensive (Andrade et al., 2015; Souza et al., 2017), vasorelaxant (Andrade et al., 2015), hypoglycemic (Hsu et al., 2016), nephroprotective (Wu et al., 2016; Romão et al., 2019), and anti-obesity (Lenquiste et al., 2019) effects. Moreover, cardioprotective effects on the doxorubicin-induced heart failure model have also been reported (Romão et al., 2019). Despite widespread usage, its antiatherosclerotic properties remain unknown. Thus, the present study aimed to investigate the cardioprotective effects of a preparation obtained from the fruit peels of *P. cauliflora* against the New Zealand rabbit’s atherosclerosis model.

## 2 Materials and methods

### 2.1 Chemicals

Isoflurane and potassium chloride were purchased from Cristália (Itapira, SP, Brazil). Simvastatin and cholesterol were obtained from Sigma-Aldrich (St. Louis, MO, United States of America). All of the other reagents were acquired in analytical grade.

### 2.2 Plant material and *Plinia cauliflora* extract obtention

*Plinia cauliflora* fruits were collected at Esperança Nova, Paraná, Brazil (latitude: −23.719864, longitude: −53.802104) in September 2017. A voucher specimen (no. 6337) is deposited in the Herbarium of the Universidade Federal da Grande Dourados (UFGD). The fruit peels were manually removed and dried for 5 days. The extract (EEPC) was made from fruit peels by using accelerated solvent extraction (Dionex^®^) with a mixture of ethanol and water (7:3, volume to volume). Nitrogen was used during the extraction process. The following parameters were used and repeated three times: a temperature of 125°C, a static extraction time of 4 min, a washing volume of 100%, a pressure of 1500 psi, and a purging time of 60 s. The EEPC was then concentrated using a rotary evaporator (Büchi R-3, Flawil, Switzerland) under reduced pressure, and finally, it was freeze-dried resulting in a yield of 30%.

#### 2.2.1 Phytochemical characterization using liquid chromatography with diode array detection and mass spectrometry (LC-DAD-MS)

A Shimadzu Prominence UFLC Shimadzu device connected to a diode array detector (DAD) and mass spectrometer MicrOTOF-Q III (Bruker Daltonics, Billerica, MA, United States of America) was used for the experiment. The column used was a Kinetex C18 column with a particle size of 2.6 µm, dimensions of 150 mm × 2.1 mm, and sourced from Phenomenex. The injection volume, flow rate, and oven temperature were 1 μL, 0.3 mL/min, and 50°C, respectively. The mobile phase consisted of a mixture of formic acid (0.1% v/v) in both water (solvent A) and acetonitrile (solvent B), using a gradient elution profile. The gradient profile used was as follows: from 0 to 2 min, 3% B; from 2 to 25 min, 3%–25% B; from 25 to 40 min, 25%–80% B; at 40–43 min, 80% B; at 43–44 min, 80%–3% B; and finally, from 44 to 48 min, 3% B. The analyses were conducted in both negative and positive ion modes. Nitrogen was used as both a nebulizer gas (at a pressure of 4 Bar) and as a dry gas (at a flow rate of 9 L/min). The capillary voltage applied was 3.5 kV. Prior to injection, the EEPC were solubilized at a concentration of 1 mg/mL, filtered, and then injected into the system.

### 2.3 Pharmacological study

#### 2.3.1 Animals

We used 14-week-old male New Zealand rabbits (1.8–2.0 kg) from Universidade Federal do Paraná (UFPR, Brazil). The animals were kept in the vivarium at Universidade Paranaense (UNIPAR) under controlled environmental conditions (temperature 20°C ± 2°C; humidity 50% ± 10%; and a 12 h/12 h light/dark cycle) with free access to food and water. The Ethics Committee of UNIPAR approved all procedures under number 35489/2019.

#### 2.3.2 Experimental procedures

The animals were initially divided into five experimental groups, with each group consisting of six animals. They were then given a standard commercial diet (Nestlé Purina PetCare, San Luis, Missouri, EUA), which was supplemented with 1% cholesterol. To prepare the CRD (cholesterol-rich diet), the commercial diet was crushed, and cholesterol dissolved in corn oil was added. After thorough mixing, the resulting mass was shaped into pellets and dried. For a period of 60 days, the CRD was freely available to the rabbit groups. Only animals with confirmed hypercholesterolemia were included in the study (Barboza et al., 2016). Thirty days after starting the diet, different experimental groups received orally, once a day, the EEPC (10, 30, and 100 mg/kg), vehicle (filtered water, 1 mL/kg; negative control), or simvastatin (SMV; 2.5 mg/kg; positive control) for 30 days. The naïve group was fed a cholesterol-free diet and was treated only with the vehicle.

Body weight gain, behavioral changes, and mortality rate were monitored throughout the experimental period. On the morning of the sixty-first day, all animals were fasted for 6 hours and anesthetized with isoflurane. Blood samples were obtained from the jugular vein, and serum was obtained by centrifugation (1,500 x g for 5 min). The levels of total cholesterol (TC), low-density lipoprotein cholesterol (LDL-C), high-density lipoprotein cholesterol (HDL-C), and triglyceride (TG) were measured using an automated biochemical analyzer (Roche Cobas Integra 400 plus). Malondialdehyde (MDA) levels were measured using an MDA assay kit (Cayman Chemical, Ann Arbor, MI, United States of America). Plasma nitrite levels were determined by the technique described by Schmidt et al. (1989). Interleukin-1β (IL-1β), IL-6, soluble vascular cell adhesion molecule-1 (sVCAM-1), soluble intercellular adhesion molecule-1 (sICAM-1), nitrotyrosine (NT), and serum-oxidized low-density lipoprotein (ox-LDL) levels were measured by enzyme-linked immunosorbent assay (ELISA; BD Biosciences, San Jose, CA, United States of America). Subsequently, all animals were euthanized (35 mg/kg potassium chloride, i.v.). A part from the aorta segments, including the arch and iliac branches, was removed and fixed in 10% formalin. After 48 h, a part of each arterial branch was stained in Sudan-IV according to the previously described method (Gasparotto et al., 2019). The luminal surface was assessed for sudanophilic lesions by Motic Images Plus 2.0 software. The edited image was classified using an iterative algorithm for multiple threshold detection. A second part of the arterial branches was dehydrated, embedded in paraffin, and sectioned at 5 μm. Then, the samples were stained with hematoxylin/eosin and microscopically examined. The images were obtained, and the intima and media layers were measured by Motic Images Plus 2.0 software, according to Gasparotto et al. (2019). Finally, a third part of the arterial branches was sectioned and homogenized in K+ phosphate buffer (0.1 M, pH 6.5). The superoxide dismutase (SOD) (Gao et al., 1998) and catalase (CAT) (Aebi, 1984) levels were measured.

### 2.4 Statistical analysis

The results are the mean ± standard deviation (n = 6 per group). The statistical analyses were performed using a one-way analysis of variance (ANOVA) followed by Bonferroni’s test. A *p* < 0.05 value was considered statistically significant. The graphs were drawn, and the statistical analysis was carried out using GraphPad Prism 9 for macOS (San Diego, CA, United States).

## 3 Results

### 3.1. Phytochemical characterization by LC-DAD-MS

**FIGURE 1:**
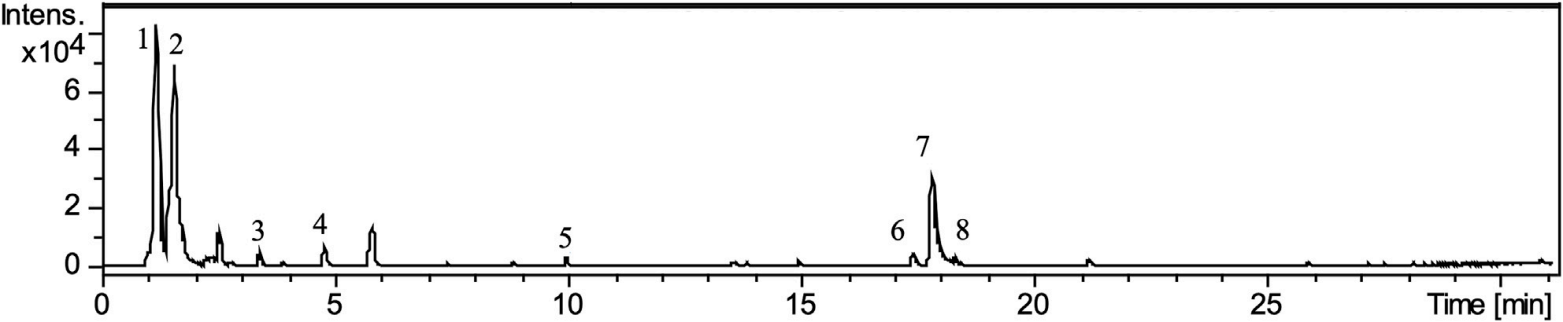
Total ion chromatogram (negative ion mode) from the EEPC.

The EEPC chromatographic fingerprinting obtained by LC-DAD-MS is presented in Figure 1 **.** The content of phenolic compounds and tannins in EEPC was 299.60 ± 4.26 (mg gallic acid equivalent [GAE] g-1) and 179.46 ± 1.76 (mg GAE g-1), respectively. The main representatives identified by LC-DAD-MS were quinic acid (1), citric acid (2), castalagin (3 and 4), vesticalagin (5), o-pentosyl ellagic acid (6), ellagic acid (7), and deoxyhexosyl ellagic acid (8).

### 3.2 Clinical and behavior parameters

**FIGURE 2:**
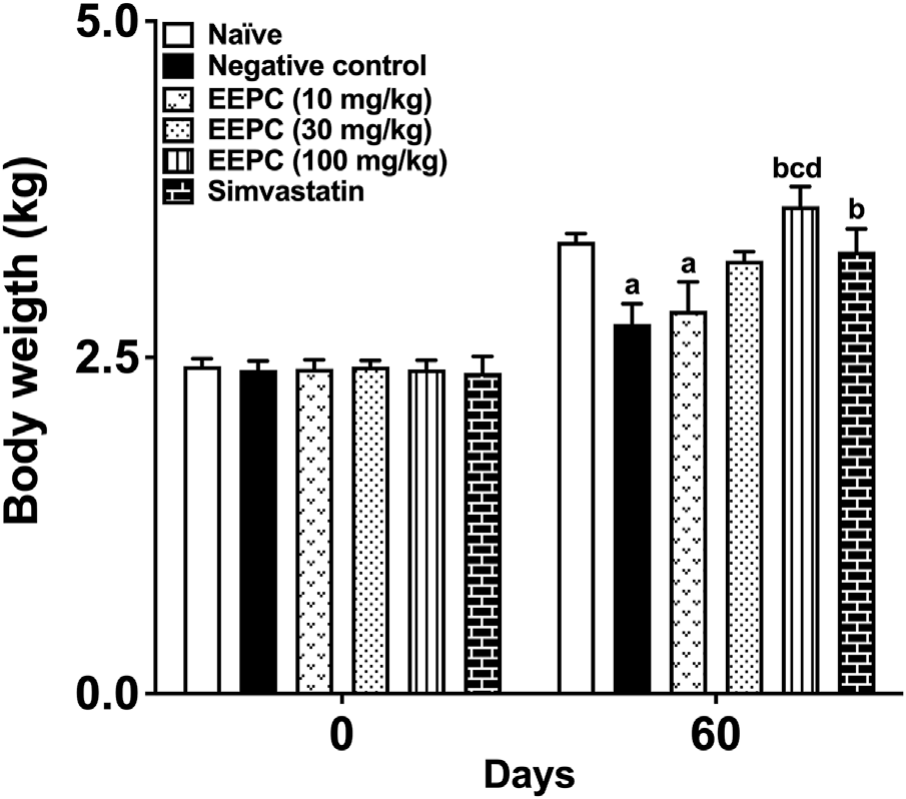
EEPC treatment prevents a reduction in weight gain in atherosclerotic rabbits. Body weight was measured at the beginning of experiments (day 0) and after 60 days of treatment with EEPC (10, 30, and 100 mg/kg), simvastatin (2.5 mg/kg), or vehicle (1 mL/kg). Values are expressed as mean ± S.E.M. (n = 6) in comparison with naïve (^a^p < 0.05), negative control (^b^< 0.05), EEPC 10 mg/kg (^c^p < 0.05), or EEPC 30 mg/kg (^d^p < 0.05) using one-way ANOVA followed by Bonferroni’s test.

The body weight gain of the different experimental groups is presented in Figure 2. At the beginning of the treatments, the body weight of the rabbits of all experimental groups was homogeneous. The animals in the negative control and EEPC 30 mg/kg groups showed a significant reduction in weight gain at the end of the 60 days of treatment. On the sixty-first day, the animals treated with EEPC at 100 mg/kg and the rabbits treated with simvastatin presented a final body weight similar to that found in naïve animals. During the experimental period, we did not identify deaths or significant behavioral changes, with appearance (skin, eyes, and appendages), reflexes, walking, and gastrointestinal function within the normal range for the species and gender.

### 3.3 Serum lipids

**FIGURE 3:**
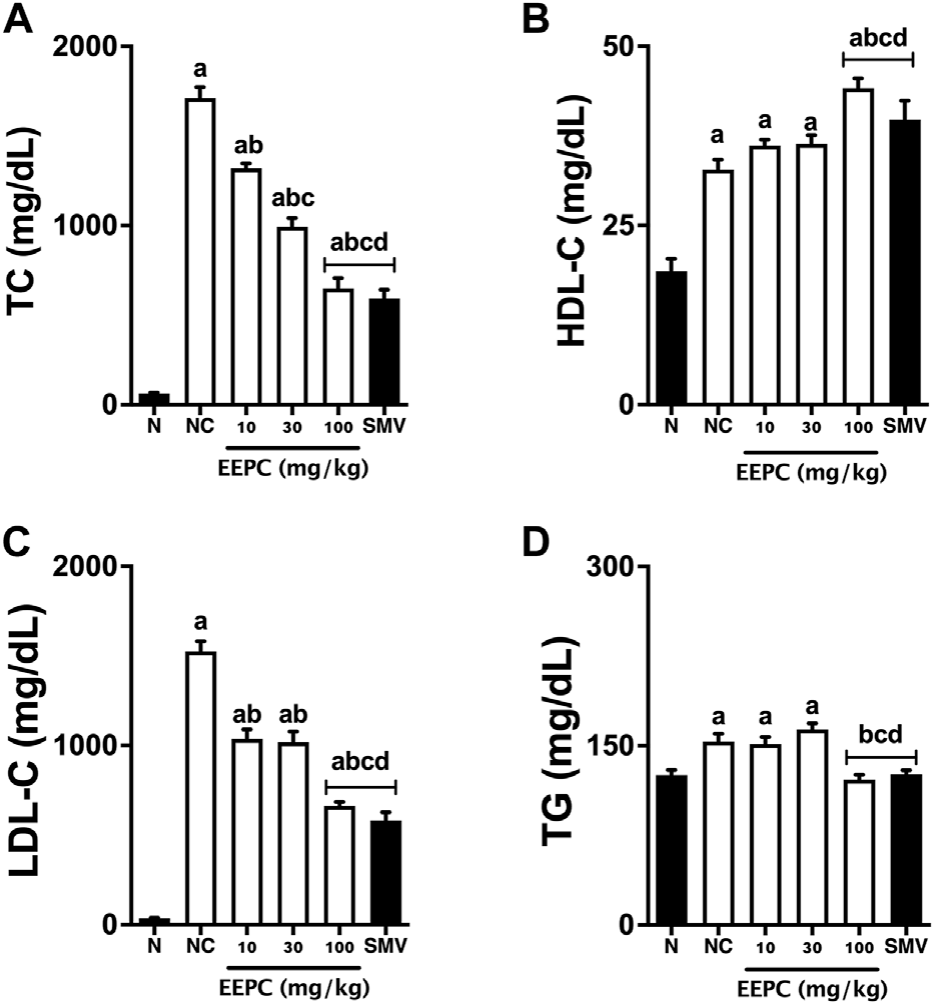
EEPC treatment reduces lipid serum levels of atherosclerotic rabbits. Serum samples were obtained on the morning 61st day of experiments, and TC **(A)**, HDL-C **(B)**, LDL-C **(C)**, and TG **(D)** levels were analyzed. Values are expressed as mean ± S.E.M. (n = 6) in comparison with naïve (^a^p < 0.05), negative control (^b^p < 0.05), EEPC 10 mg/kg (^c^p < 0.05), or EEPC 30 mg/kg (^d^p < 0.05) using one-way ANOVA followed by Bonferroni’s test. HDL-C: high-density lipoprotein cholesterol; LDL-C: low-density lipoprotein cholesterol; N: naïve group; NC: negative control group; SMV: simvastatin; TC: total cholesterol; TG: triglyceride.

Serum TC, HDL-C, LDL-C, and TG levels from all experimental groups are shown in Figures 3A–D. Serum levels of TC, HDL-C, LDL-C, and TG in naïve animals were found to be 63 ± 9 mg/dL, 18 ± 4.2 mg/dL, 33 ± 8 mg/dL, and 125 ± 10 mg/dL, respectively, after 60 days of the experiment. The negative control group showed a significant increase in the levels of all measured serum lipids, with values of 1,712 ± 149 mg/dL, 32 ± 3.5 mg/dL, 1,524 ± 139 mg/dL, and 153 ± 16 mg/dL for the TC, HDL-C, LDL-C, and TG levels, respectively. Oral EEPC treatment was able to reduce the serum TC and LDL-C dose-dependently. At the highest dose, EEPC induced a lipid-lowering effect similar to simvastatin treatment. The high-cholesterol diet increased HDL-C levels in all experimental groups, showing significantly higher values in the EEPC 300 mg/kg and simvastatin groups. Triglyceride levels were significantly elevated in the negative control and EEPC 10 and 30 mg/kg groups. In rabbits treated with EEPC 100 or simvastatin, TG levels were similar to those found in naïve animals.

### 3.4 Redox state

**FIGURE 4:**
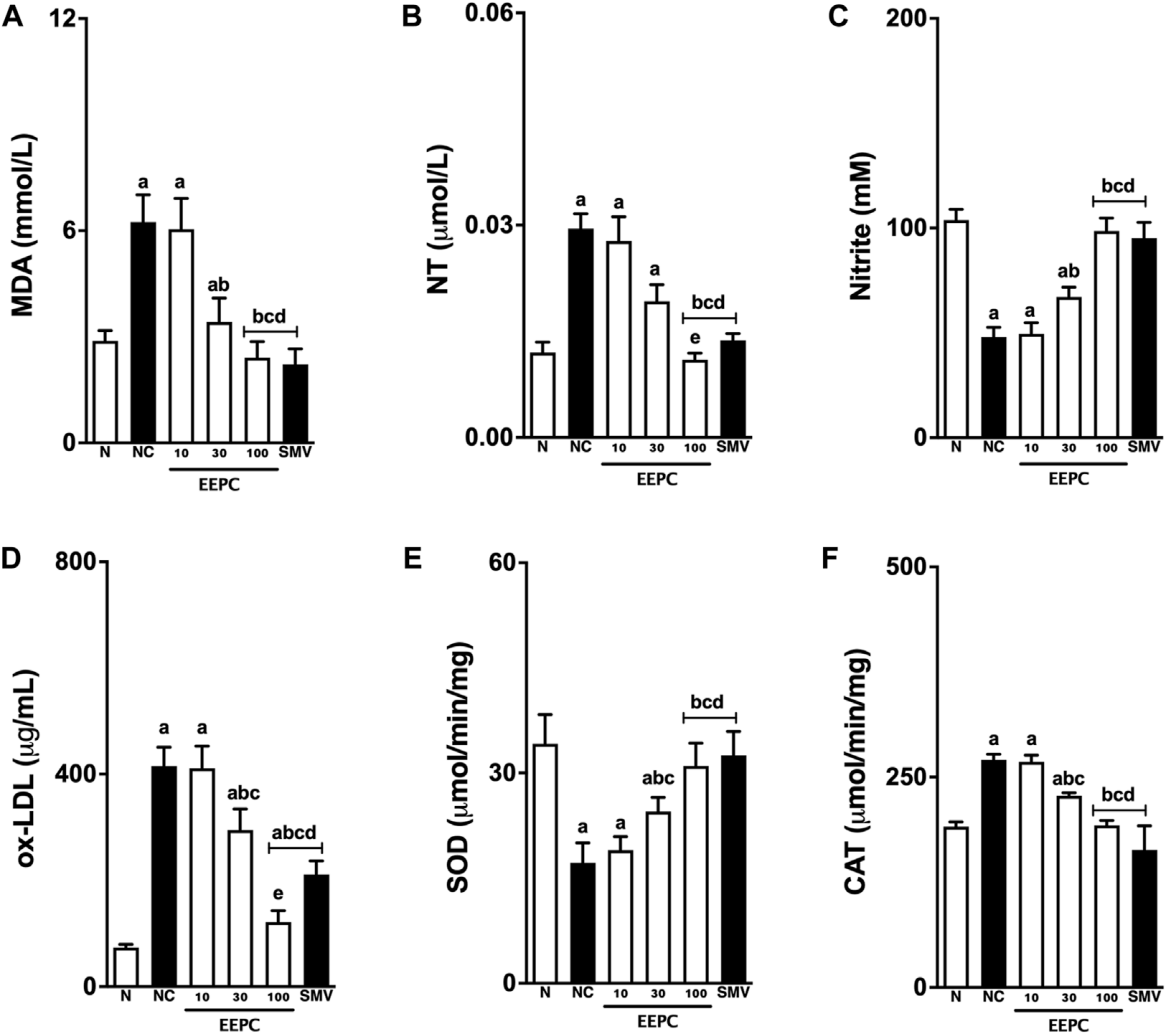
EEPC treatment modulates the redox state of atherosclerotic rabbits. Malondialdehyde (MDA; **(A)**, nitrotyrosine (NT; **(B)**, serum nitrite **(C)**, oxidized low-density lipoprotein (Ox-LDL; **(D)**, superoxide dismutase (SOD; **(E)**, and catalase (CAT; **(F)** are presented. Values are expressed as mean ± S.E.M. (n = 6) in comparison with naïve (^a^p < 0.05), negative control (^b^p < 0.05), EEPC 10 mg/kg (^c^p < 0.05), or EEPC 30 mg/kg (^d^p < 0.05) using one-way ANOVA followed by Bonferroni’s test. N: naïve group; NC: negative control group; SMV: simvastatin.

The levels of different redox state serum markers are shown in Figures 4A–D. Negative control animals exhibited significant increases in MDA and NT serum levels (∼70%) compared with naïve animals. Similarly, ox-LDL serum levels increased from 76 ± 6 μg/mL in the naïve group to 415 ± 35 μg/mL in the negative control rabbits. Treatment with EEPC (30 and 100 mg/kg) significantly reduced TBARS and NT levels, and at its highest dose (300 mg/kg), the response was to values that were close to naïve animals. Similarly, the EEPC treatment (30 and 100 mg/kg) also significantly reduced the ox-LDL levels, and at its highest dose (300 mg/kg), the response was lower than that found in animals that received SMV. On the other hand, nitrite serum levels were significantly (∼60%) reduced in animals from the negative control group. Treatment with EEPC was able to increase nitrite levels significantly. When treatment was carried out with the highest dose of EEPC (100 mg/kg), nitrite values were similar to those obtained in the SMV group and naïve animals.

The analysis of the antioxidant enzymes present in the arterial tissue of the different experimental groups is shown in Figures 4E, F. The atherogenic diet decreased the SOD levels by ∼ 60% and increased the CAT arterial levels by ∼ 30% in negative control animals. Treatment with EEPC (30 and 100 mg) attenuated these alterations in all of the evaluated arterial samples. At its highest dose, EEPC maintained SOD and CAT values similar to those in naïve or SMV-treated animals.

### 3.5 Inflammatory markers

**FIGURE 5:**
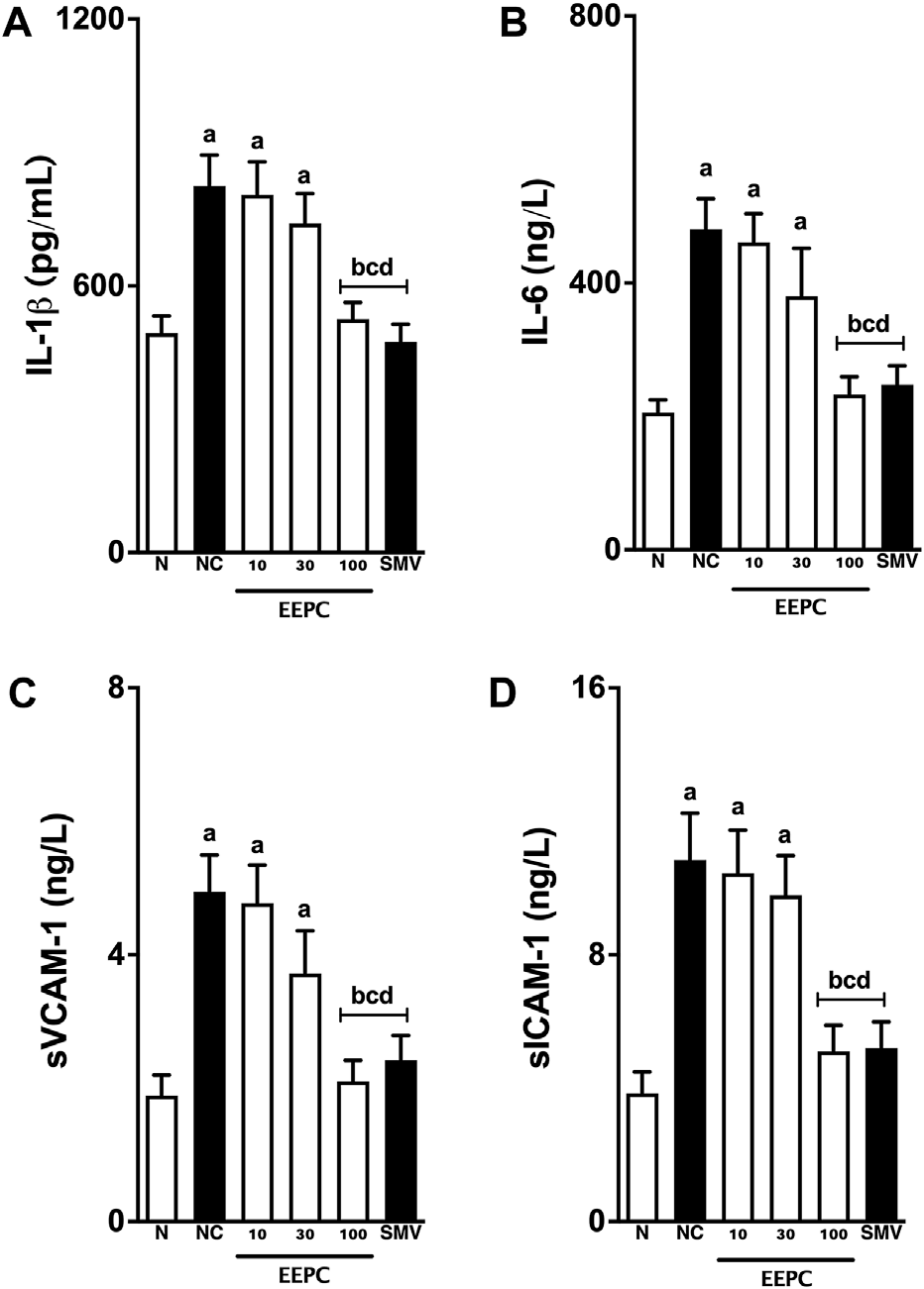
EEPC treatment reduces interleukins and soluble adhesion molecules levels in atherosclerotic rabbits. Serum L-1β **(A)**, IL-6 **(B)**, sVCAM-1 **(C)**, and sICAM-1 **(D)** levels are shown. Values are expressed as mean ± S.E.M. (n = 6) in comparison with naïve (^a^p < 0.05), negative control (^b^p < 0.05), EEPC 10 mg/kg (^c^p < 0.05), or EEPC 30 mg/kg (^d^p < 0.05) using one-way ANOVA followed by Bonferroni’s test. N: naïve group; NC: negative control group; SMV: simvastatin.

Interleukin-1β, IL-6, sVCAM-1, and sICAM-1 serum levels in naïve and atherogenic rabbits are shown in Figures 5A–D. Baseline levels of IL-1β, IL-6, VCAM-1, and sICAM-1 in the naïve animals were 493 ± 38 pg/mL, 205 ± 19 ng/L, 1.8 ± 0.3 ng/L, and 3.8 ± 0.6 ng/L, respectively. Consumption of an atherogenic diet for 60 days significantly increased this parameter in animals from the negative control group, with values estimated at 824 ± 69 pg/mL, 480 ± 46 ng/L, 4.9 ± 0.5 ng/L, and 10 ± 1.4 ng/L for IL-1β, IL-6, sVCAM-1, and sICAM-1, respectively. The treatment with EEPC 100 mg/kg reduced IL-1β, IL-6, sVCAM-1, and sICAM-1 serum levels to values close to naïve rabbits and simvastatin-treated animals.

### 3.6 Histopathological finds

**FIGURE 6:**
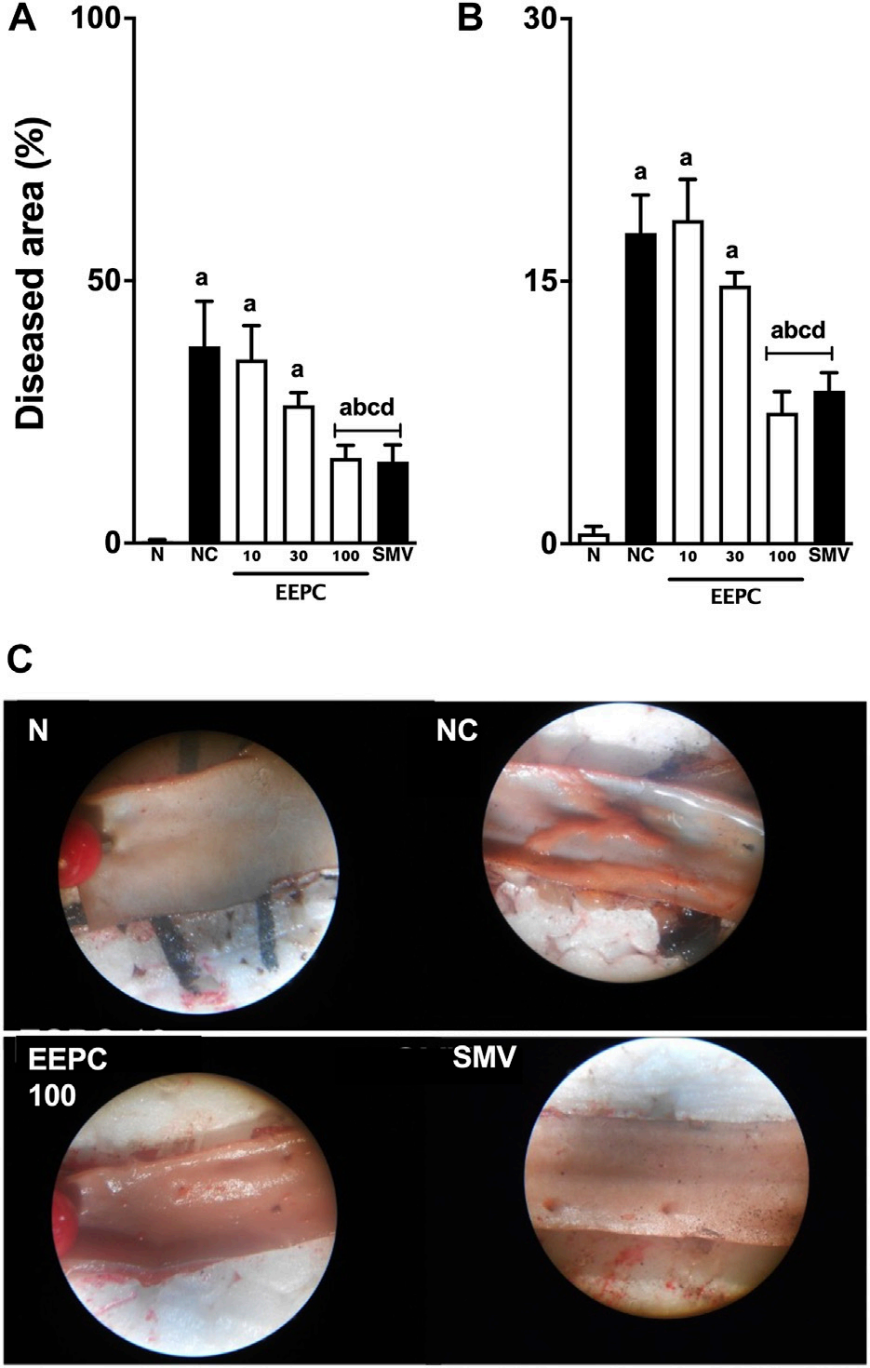
EEPC treatment reduces sudanophilic lesions in aorta segments of atherosclerotic rabbits. The average area of atherosclerotic lesions in the aortic arch **(A)**, iliac segments **(B)**, and representative macroscopic observations in the aortic arch **(C)** are shown—images in a 4-X scale. Values are expressed as mean ± S.E.M. (n = 6) in comparison with naïve (^a^p < 0.05), negative control (^b^p < 0.05), EEPC 10 mg/kg (^c^p < 0.05), or EEPC 30 mg/kg (^d^p < 0.05) using one-way ANOVA followed by Bonferroni’s test. N: naïve group; NC: negative control group; SMV: simvastatin.

The macroscopic lesions observed in the aortic arch and iliac branch are shown in Figures 6A–C. In the negative control group, the total average area of the aortic arch and aortoiliac branches were 37 ± 8 mm^2^ and 17 ± 2 mm^2^, respectively. The average area of sudanophilic lesions in the aortic arch and aortoiliac segments in the EEPC 100 mg/kg treated rabbits was decreased by ∼ 57 ± 6% and 59% ± 9%, respectively. The EEPC 100 mg/kg effects were statistically similar to the standard drug simvastatin.

**FIGURE 7:**
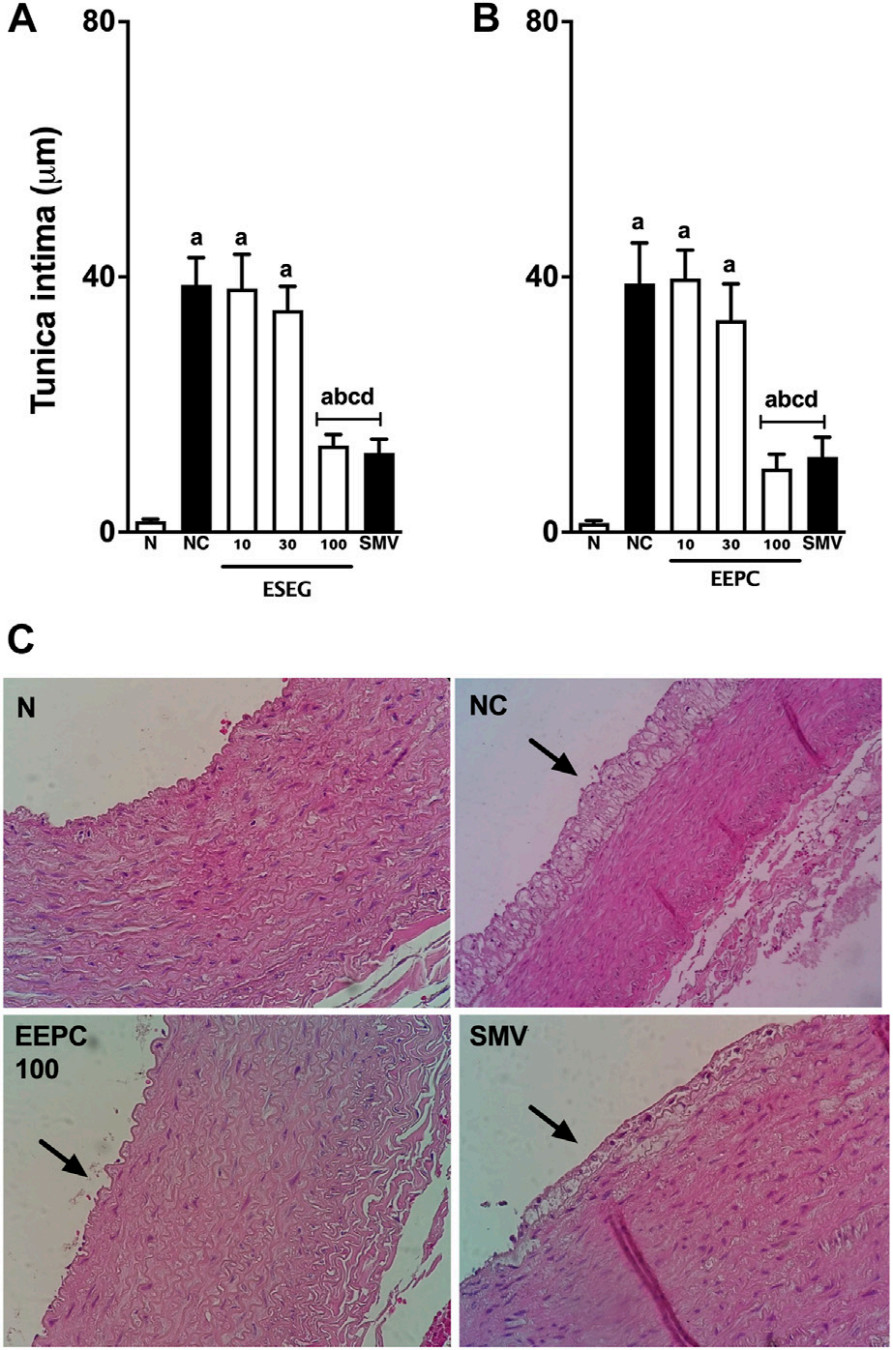
EEPC treatment reduces arterial thickness in aorta segments of atherosclerotic rabbits. Histomorphometric analyses of tunica intima from the aortic arch **(A)** and iliac segments **(B)** are shown. Representative cross-sections of the aortic arch stained with hematoxylin-eosin are also presented **(C)**—images in 40-X scale. Values are expressed as mean ± S.E.M. (n = 6) in comparison with naïve (^a^p < 0.05), negative control (^b^p < 0.05), EEPC 10 mg/kg (^c^p < 0.05), or EEPC 30 mg/kg (^d^p < 0.05) using one-way ANOVA followed by Bonferroni’s test. N: naïve group; NC: negative control group; SMV: simvastatin.

Morphometric measures of the intima layer of the aortic arch and iliac segment are shown in Figures 7A–C. The average estimate of aorta segments and iliac branch in the positive control group showed significant thickness in the intima layer (aortic arch: 38 ± 4 μm; iliac branch: 39 ± 6 μm) compared with the naïve rabbits. Treatment with ESEG (100 mg/kg) reduced the thickness of the intima layer of aorta segments and iliac branch by ∼ 65 ± 7% and 74% ± 8%, respectively, with values very similar to animals that were treated with simvastatin. No significant morphometric differences in the media layers were observed among groups (data not shown).

## 4 Discussion

Herbal medicines and phytochemicals were essential sources for the advancement of medicine and continue to play an important role in developing new drugs and as an alternative and complementary treatment for a large part of the world’s population (Falzon and Balabanova, 2017). In Brazil, *Plinia cauliflora* is an important example, as it has been previously stated to have numerous pharmacological activities (Gasparotto Junior et al., 2019). With the objective of investigating the effects of *P. cauliflora* extract on the progression of atherosclerotic disease, we chose to use the classic atherosclerosis model, which involved feeding rabbits with a diet rich in cholesterol (Santiago et al., 2017; Gasparotto et al., 2018; Gasparotto et al., 2019). Currently, rabbits are considered an important pre-clinical model for studying lipoprotein metabolism and atherosclerosis. This model is typically based on inducing hypercholesterolemia through HFD, which eventually lead to the development of atherosclerosis. The presence of atherosclerosis can be observed after only 1 week of exposure (Emini Veseli et al., 2017). Rabbits and humans share certain lipid characteristics that are not seen in rodents. These include a higher abundance of LDL-C particles in the bloodstream, activity of the LDL-C receptor in the liver, the presence of VLDL-C receptors on macrophages, the cholesterol ester transferase protein (CETP), heterogeneous HDL-C particles, and sensitivity to dietary cholesterol (Emini Veseli et al., 2017).

World Health Organization points out that cardiovascular diseases (CVD) are the leading causes of morbidity and mortality worldwide, with values estimated at 17.9 million deaths annually (Nugroho et al., 2022). Among CVD, atherosclerosis contributes a large part to this outcome. Atherosclerosis is a complex inflammatory disease that affects medium and large-caliber arteries, especially in areas of more significant shear stress or bifurcations. The speed of its evolution depends on several factors, especially the intake of a diet rich in fats, hypertension, smoking, diabetes, and family history (Libby et al., 2019). Epidemiological studies indicate that high levels of LDL-C play a central role in the development of atherosclerosis (Whayne, 2017). Preclinical data and population studies show that endothelial dysfunction and increased LDL-C in its oxidized form (OxLDL) are indeed the primary cause of atherosclerosis. The morphological change in endothelial cells, followed by increased permeability to OxLDL particles in the subendothelial space, is the kickstart of atherosclerosis (Pirillo et al., 2013).

One of the characteristics of the experimental model used in this study is the high ease with which rabbits develop significant dyslipidemia, notoriously LDL-C. Thus, we could effectively mimic the limiting stage of the atherosclerotic process developed in humans. EEPC was notoriously effective in reducing blood lipid levels, especially LDL-C, pointing to a probable mechanism for the prevention evolution of the atherosclerotic disease. Several experimental studies have already linked the hypolipidemic effects of herbal medicines and phytochemicals, especially phytocomplexes or compounds-like flavonoids, polyphenols, and polysaccharides (Liu et al., 2022). Furthermore, the concept of synergistic hypolipidemic is rapidly increasing as a practical approach to the health-promoting effects of functional ingredients. Researchers worldwide have broadened their understanding of how natural products affect absorption, synthesis, and regulating lipid transport and metabolism in recent years. Potential pharmacological targets include LDL receptor protein, Niemann-pick protein C1, 3-hydroxy-3-methylglutaryl coenzyme A reductase, ATP-binding cassette protein A1, ATP citrate lyase, sterol-regulatory element binding protein 2, peroxisome proliferator-activated receptor, cholesterol-7α-hydroxylase, cholesteryl ester transfer protein, acetyl-CoA carboxylase, 5′-monophosphate-activated protein kinase, fatty acid synthetase, carbohydrate responsive element-binding protein, carnitine palmitoyltransferase1A, and adipose triglyceride lipase, hormone-sensitive lipase (Momtazi-Borojeni et al., 2019).

Reactive oxygen species (ROS) are produced by cellular metabolism. Under certain conditions, including dyslipidemia, cells will overproduce ROS, and several response mechanisms will be activated, including enzymatic antioxidants such as SOD and CAT. SOD primarily metabolizes superoxide anion into hydrogen peroxide and molecular oxygen, while CAT neutralizes excess hydrogen peroxide (Sies, 2015). Excessive production of ROS can alter cell homeostasis, leading to a chronic inflammatory response and contributing to the genesis of numerous chronic diseases, including atherosclerosis (Kattoor et al., 2017). It is already widely known that oxidative stress reduces nitric oxide (NO) production, impairs vasodilation, and causes endothelial dysfunction (Incalza et al., 2018). Moreover, oxidative stress triggers oxidative changes that can occur in the LDL-C molecule. After binding to proteoglycans of the extracellular matrix, the OxLDL induces the release of cytokines and the expression of cell adhesion molecules on the endothelial cells, recruiting monocytes and T lymphocytes to the inflamed arterial area. Differentiation of monocytes into macrophages express scavenger receptors to recognize OxLDL. The atherosclerotic plaque is mainly formed of foam cells, originating from the metamorphosis of swollen macrophages from OxLDL molecules. Finally, the plaque is covered by a fibrous cap, which can also be affected by oxidative stress. ROS can degrade the fibrous wall of plaque via the release of matrix metalloproteinases, causing thrombus formation, blockage of blood flow, and tissue infarction (Kattoor et al., 2017). We showed that EEPC could attenuate the oxidative stress induced by the atherogenic diet, reducing protein and lipid peroxidation and increasing nitrite levels, an indirect marker of nitric oxide bioavailability. This effect likely has an essential contribution from tissue antioxidant enzymes, especially SOD and CAT, as prolonged treatment with EEPC modulated the presence of these enzymes in different arterial branches. The evidence points to a synergistic effect between the reduction of serum lipids and antioxidant activity, reverberating in a significant decrease in LDL-C oxidation and the release of inflammatory mediators, including IL-1β, IL-6, sVCAM, and sICAM. As an endpoint, we found a significant reduction in lipid streaks and mature atherosclerotic plaques in all evaluated arterial branches.

The antioxidant potential of natural products is already well known, mainly due to the polyphenols (phenolic acids, flavonoids, anthocyanins, lignans, and stilbenes), carotenoids (xanthophylls and carotenes) and vitamins (vitamin E and C) (Lu et al., 2021). Generally, these natural antioxidants, especially polyphenols, exhibit a wide range of cardiovascular effects, such as in both animal models and clinical trials (Zhang et al., 2021). In recent years, several studies have explored the antiatherosclerotic effects of natural products in New Zealand rabbits and have associated polyphenolic compounds as potential agents responsible for the cardioprotective effects. In our study, we identified a large number of polyphenolic compounds in EEPC, including organic acids, phenolic acid derivatives, flavonoids, anthocyanins, and hydrolysable tannins. These findings indicate that these compounds are likely to contribute to the presented antiatherosclerotic response. However, it would be highly speculative to attribute the antiatherosclerotic activity of EEPC solely to one compound, like a flavonoid. We believe that the cardioprotective effects occur due to a synergistic and coordinated activity of different polyphenolic molecules, which contribute directly to the observed dose-response effect.

Despite the promising results, our study brought to light two limitations. First, we could not identify the molecular mechanism responsible for hypolipidemic effects induced by EEPC. Second, if the antioxidant effect is due to the modulating response on antioxidant enzymes or even the free radical scavenging effect of the polyphenolic compounds present in the EEPC. Future studies may clarify these points and open the perspective of using EEPC as a cardioprotective herbal medicine.

## 5 Conclusion

In conclusion, our results confirm that EEPC prevented the evolution of high-cholesterol diet-induced atherosclerosis in rabbits. *Plinia cauliflora* extract improved lipid profile and restored antioxidant enzymes in atherosclerotic rabbits, and it also reduced vascular dysfunction by restoring NO levels in endothelial cells. These findings indicate that EEPC can be a potential candidate for add-on management of atherosclerosis.

## Data Availability

The raw data supporting the conclusion of this article will be made available by the authors, without undue reservation.
